# The Effect of Alloying Elements on the Structural Stability, and Mechanical and Electronic Properties of Al_3_Sc: A First-Principles Study

**DOI:** 10.3390/ma12091539

**Published:** 2019-05-10

**Authors:** Dong Chen, Cunjuan Xia, Xiaomin Liu, Yi Wu, Mingliang Wang

**Affiliations:** 1 State Key Laboratory of Metal Matrix Composites, Shanghai Jiao Tong University, No. 800 Dongchuan Road, Shanghai 200240, China; chend@sjtu.edu.cn (D.C.); xiacunjuan@sjtu.edu.cn (C.X.); 2 School of Materials Science & Engineering, Shanghai Jiao Tong University, No. 800 Dongchuan Road, Shanghai 200240, China; eagle51@sjtu.edu.cn

**Keywords:** ab-initio calculations, structural stability, elastic properties, electronic properties

## Abstract

The first-principles methods, based on the density function theory, are performed to calculate the properties of pure and doped Al_3_Sc. The structural stability, and mechanical and electronic properties of L1_2_-Al_3_Sc_1−*x*_M*_x_* (M = Zr, Ti, Y, and Li) have been investigated. A negative formation enthalpy for L1_2_-Al_3_Sc_1–*x*_M*_x_* indicated that all doped structures were stable, and Al_24_Sc_6_Zr_2_ was found to be the most stable. The elastic constants, elastic moduli and Debye temperatures of Al_3_Sc, with different doping elements and different doping concentrations, were calculated to explore the influences of doping on the mechanical properties and Debye temperatures of Al_3_Sc. Furthermore, the calculated results suggested that both Al_24_Sc_6_Zr_2_ and Al_24_Sc_6_Ti_2_ could optimize the mechanical properties. Finally, the electronic properties based on the analyses of densities of states and electron density distributions, have been performed, to explain the underlying mechanisms for the structural and mechanical properties of the L1_2_-Al_3_Sc_1–*x*_M*_x_* structures.

## 1. Introduction

In the past decades, Al-Sc alloys have attracted great attention, due to higher strength and stiffness, compared with pure Al [1,2]. The addition of Sc can greatly improve the mechanical properties of Al alloy, since a large number of nanoparticles (Al_3_Sc) are formed in the aging process [3,4,5], and Al_3_Sc with a cubic L1_2_ structure has a small lattice mismatch with α-Al. However, the industrial application of Al_3_Sc has been limited because of the high cost of Sc, and the low solubility of Sc in Al. As a result, it is necessary to find an element to replace Sc, which can improve the mechanical properties of the alloy and reduce its cost.

In recent years, researcher has shown that doping has a certain impact on the structural characteristics and mechanical properties of alloys, through experimental investigations [6,7,8,9] and theoretical calculations [10,11,12]. The behaviors and properties of the alloying elements in Al_3_Sc, have been studied by experiments. For example, Fuller et al. studied the replacement of Sc by Zr in Al-Sc alloys, and found that the coarsening resistance was increased at higher temperatures [13]. Dalen et al. studied the effects of Ti additions on the structural and creep properties of Al-Sc alloys [14]. Seidman et al. indicated that the addition of Li has resulted in an increased driving force for precipitate nucleation [15]. Harada et al. compared the thermal expansion and creep properties of Al_3_Sc and Al_3_(Sc, Y) [16,17]. Up to now, the microstructure, compression, fracture behavior, elastic and optical properties of Al_3_Sc have been widely researched [18,19] by the density functional theory(DFT) method. Furthermore, the structural, electronic, mechanical, and thermodynamic properties of Al_3_Sc, under different pressures and temperatures, have been calculated [20,21,22]. Moreover, the effect of transition metals on the structural stability of Al_3_Sc_1-x_M_x_ was studied by the special quasi random structures method [23]. In addition, the properties of Al_3_Sc_1−*x*_M*_x_* (M=Zr, Ti) with different concentrates, were performed [24,25]. Nevertheless, the influences of the doping elements on the mechanical and electronic properties of the Al-Sc-M system have required further investigations.

In this work, we calculated the structural stability and mechanical properties of the L1_2_-Al_3_Sc_1–*x*_M*_x_* (M = Zr, Ti, Y, and Li) structures with concentrations of 3.125 at.% and 6.25 at.%. Second, the mechanical properties and electronic properties of the L1_2_-Al_3_Sc_1–*x*_TM*_x_* structures have been intensively discussed in relation to their electronic properties. This investigation could provide theoretical guidance to the application of Al-Sc-based alloys.

## 2. Materials and Methods 

All calculations were performed on the basis of the density functional theory (DFT) with the Vienna Ab Initio Simulation Package (VASP) [26]. The pseudopotential in the reciprocal space was described by the projector-augmented wave (PAW) method [27]. The generalized gradient approximation (GGA), with the Perdew-Burke-Ernzerhof (PBE) [28] function was applied to describe the exchange-correlation potential. Both the k-space integral and plane-wave basis were chosen to ensure that the total energy was converged. The convergence criterion for the self-consistent field energy was set to be 5 × 10^−6^ eV/atom. For the plane wave expansion, a kinetic cutoff energy of 500 eV was considered to be sufficient. The geometry optimization was terminated when the Hellman–Feynman force on each atom was smaller than the 0.003 eV/nm. The integral in the Brillouin zone was sampled by the Monkhorst-Pack method [29], with the k-point mesh of 21 × 21 × 21 for Al_3_Sc and 11 × 11 × 11 for the 2 × 2 × 2 supercells. All calculations were carried out with the potentials for Al(3*s*^2^3*p*^1^), Li(1*s*^2^2*s*^1^), Sc(3*s*^2^3*p*^6^3*d*^1^4*s*^2^), Ti(3*s*^2^3*p*^6^3*d*^2^4*s*^2^), Zr(4*s*^2^4*p*^6^4*d*^2^5*s*^2^), and Y(4*s*^2^4*p*^6^4*d*^1^5*s*^2^), as the valence electrons. Overall, all calculations were operated in 0 K, with the equivalent hydrostatic pressure.

## 3. Results

### 3.1. Structural Stability

**Figure 1:**
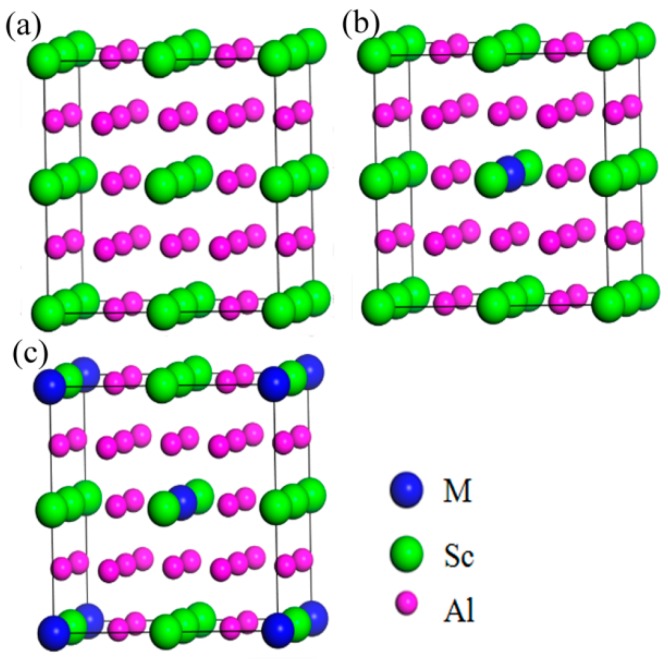
Structures of Al_3_Sc (**a**) 2 × 2 × 2 surpercell; doped with the alloying element (M = Sc, Zr, Ti, Y, Li) at different doping concentrations, (**b**) 3.125%, and (**c**) 6.25%. Blue, green, and pink balls represent (M = Zr/Ti/Y/Li), Sc, and Al atoms, accordingly.

The Al_3_Sc phase had a cubic structure (Space Group: Pm3m (No. 221)), which contained 3 Al atoms and 1 Sc atom, in a unit cell. Based on the Al_3_Sc phase, a 2 × 2 × 2 supercell (Figure 1a) was constructed to investigate the effects of the doped element (M = Zr, Ti, Y, and Li). In addition, the effects of the doping concentrations (at.%) were also considered, which included 3.125% (Figure 1b) and 6.25% (Figure 1c). The structures of the surpercells used in this work are shown in Figure 1, and the optimized structural parameters are listed in Table 1.

It is well-known that a lower formation energy implies a more stable structure. In order to study the influence of the doped elements on the structural stability, the enthalpies of formation (Δ*H_f_*) of the Al_3_Sc structure, before and after doping, were calculated with the following formula:

(1) $$\Delta H_{f}=\frac{1}{n}\left(E_{total}-N_{Al}E_{Al}-N_{Sc}E_{Sc}-N_{M}E_{M}\right)$$

where *E_total_* is the total energy of the doped structure; n stands for the total number of atoms in the Al-Sc-M system; *E_i_* and *N_i_* are the energies per atom of species *i*, and the corresponding number of atoms in the doped structure. The energies per atom of Al, Sc, and M (M = Zr/Ti/Y/Li) were calculated from bulk Al with an FCC structure, a bulk Sc and M (M = Zr/Ti/Y) with an HCP structure, and a bulk M (M = Li) with a BCC structure.

The calculation results of ΔH*_f_* are listed in Table 1. The negative ΔH*_f_* indicates that the doped structure could stably form at 0 K; all structures were stable. However, the ΔH*_f_* values of the doped structures were greater than that of pure Al_3_Sc, except for the Al-Sc-Zr system. This meant that Zr doping could improve stability, while the Ti/Y/Li dopings might reduce the structural stability. This was consistent with results from previous studies [23,30]. In addition, it was found that the stability of Al_24_Sc_6_Zr_2_ was higher in comparison to Al_24_Sc_7_Zr. Except for the Zr addition, the higher concentration dopings should have caused a lower stability of the structure.

### 3.2. Elastic Properties

The elastic properties could provide necessary information on the resistance of the material to extrinsically applied stress. For the cubic crystals, there were three independent elastic constants (i.e., C_11_, C_12_, and C_44_). The calculated results of the elastic constants are listed in Table 2. 

Notably, the results of Al_3_Sc were close to the published DFT calculations [15] and experimental measurements [31], implying the reliability of the calculation results. The criterions for the mechanical stability of the cubic crystal [32] were estimated using Equation (2):

(2) $$C_{11}-C_{12}>0,\;C_{11}>0,\;C_{44}>0,\;C_{11}+2C_{12}>0$$

The calculated results showed that the elastic constants of all structures could satisfy the above stability conditions, indicating that all structures had mechanical stability.

**Figure 2:**
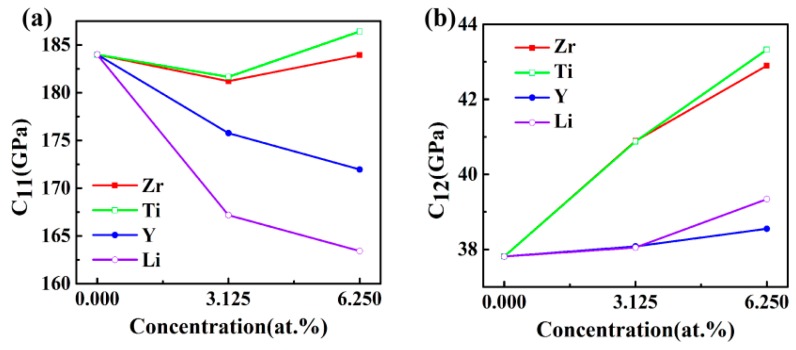

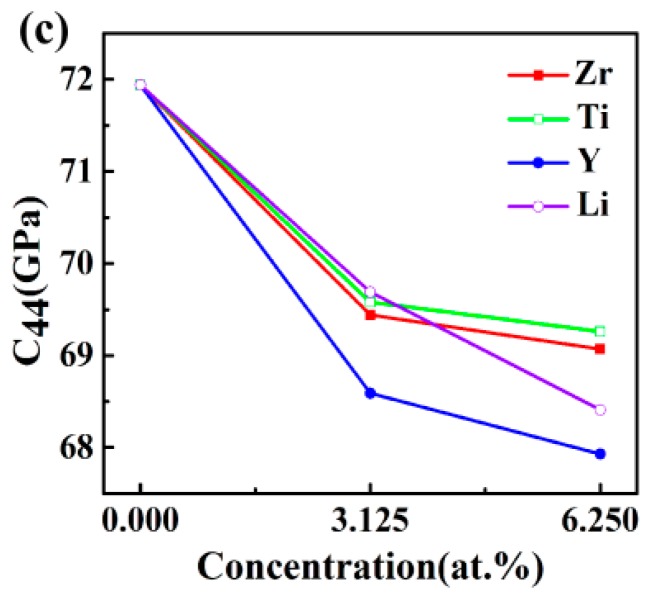
The elastic constants C_ij_ (GPa) of L1_2_-Al_3_Sc doped with the element M (M = Zr, Ti, Y, and Li) on the dependence of doping concentration: (**a**) C_11_, (**b**) C_12_, and (**c**) C_44_.

The calculated elastic constants as a function of doping concentration for Al_3_Sc are exhibited in Figure 2. The C_11_ values of Al_3_Sc, before and after doping, were larger than the other elastic constants (Figure 2a), suggesting that the axes compression resistances were stronger. The C_11_ values in the Al_24_Sc_7_M system were obviously decreased, which proved that the axes pressure resistances were reduced. However, Al_24_Sc_6_M_2_ have found to be quite complex. For example, Al_24_Sc_6_Y_2_ and Al_24_Sc_6_Li_2_ have smaller C_11_ values, Al_24_Sc_6_Zr_2_ had a similar C_11_ value, and Al_24_Sc_6_Ti_2_ possessed a larger C_11_ value. On the other hand, both Al_24_Sc_7_M and Al_24_Sc_6_M_2_ exhibited higher C_12_ values (Figure 2b), suggesting that the Poisson effect was enhanced [25]. Additionally, the Al-Sc-M systems had shown a slightly reduced tendency for C_44_ (Figure 2c), especially for the Al_24_Sc_6_M_2_, indicating that the resistance to shear deformation was gently diminished.

The polycrystalline elastic moduli are the important performance parameters of engineering materials, such as bulk modulus (*B*), shear modulus (*G*), Young’s modulus (*E*), and Poisson’s ratio (*ν*). The bulk modulus and shear modulus were determined using the Voigt-Reuss-Hill method [33]. For the cubic structure, the bulk modulus (*B*) and shear modulus (*G*) were calculated from Equations (3) and (4), respectively:

(3) $$B=B_{V}=B_{R}=\left(C_{11}+2C_{12}\right)/3$$

(4) $$G_{V}=\left(C_{11}-C_{12}+3C_{44}\right)/5\;G_{R}=5\left(C_{11}-C_{12}\right)C_{44}/\left[3\left(C_{11}-C_{12}\right)+4C_{44}\right]\;G=\left(G_{V}+G_{R}\right)/2$$

Young’s modulus (*E*) and Poisson’s ratio (*ν*) were determined by *B* and *G*, and their expressions are shown in Equations (5) and (6) [34]:

(5) E=9GB/(3B+G)

(6) ν=(3B−2G)/2(3B+G)

The results of the elastic moduli are listed in Table 3. It is well-known that *B* is a measure of the degree to which a material deforms under hydrostatic pressure [35]. *G* indicates the material’s resistance to shear strain [35]. *E* is a representation of the stiffness of the material [36]. 

**Figure 3:**
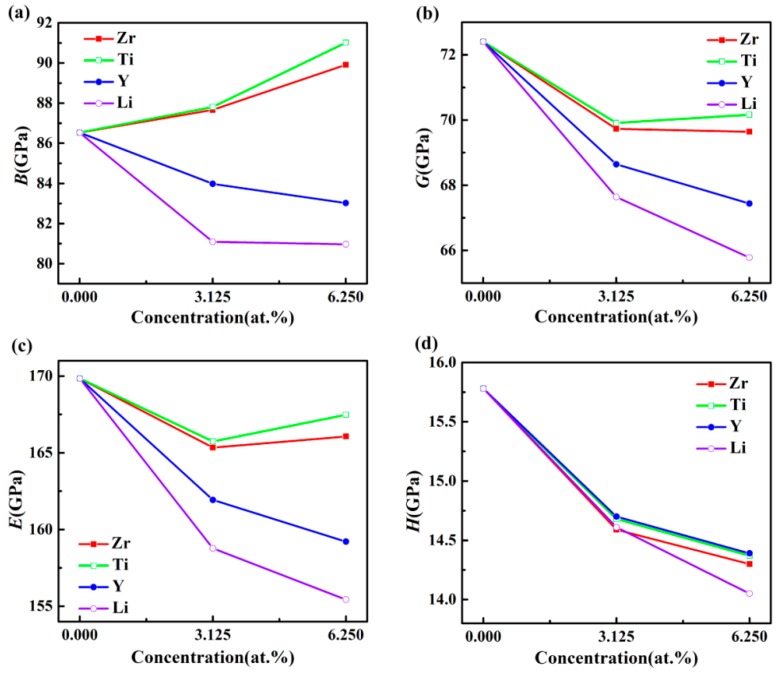
The elastic moduli *B* (**a**), *G* (**b**), *E* (**c**), and hardness *H* (GPa), (**d**) of L1_2_–Al_3_Sc doped with element M (M = Zr, Ti, Y, and Li), as a function of the doping concentration.

The influences of doped elements at different concentrations on the elastic modulus of Al_3_Sc are displayed in Figure 3a–c. The values of *B* doped with Zr and Ti increased with a growing doping concentration, while the values of *B* doped with Y and Li decreased with an increasing doping concentration. When the doping concentration was constant, Ti doped Al-Sc-M compounds had the maximum *B* values, implying that the addition of Ti into Al_3_Sc resulted in minimal deformation, under certain external pressure.

Furthermore, the doped Al-Sc-M compounds displayed lower *G* and *E*, meaning that the shear strain resistance and stiffness were reduced. In addition, the *E* values of Al_3_Sc doped with Zr and Ti were increased, when the doping concentrations increased from 3.125% to 6.25%, indicating that the higher concentration doping showed a better performance for the Al-Sc-M (M = Zr/Ti). 

The investigation of the stiffness could be completed by providing the microhardness parameter (*H*), given by the following relation [37]:

(7) $$H=\frac{\left(1-2\upsilon \right)}{6\left(1+\upsilon \right)}E$$

Figure 3d exhibits the *H* values of Al_3_Sc with different doping elements and concentrations. It is clearly observed that the *H* values of the doped Al_3_Sc decreased with an increasing concentration. For example, Al_24_Sc_6_Ti_2_ presented the maximum *H* value. Although the *H* values of the doped Al_3_Sc were decreased, the reduced magnitude was small, compared with that of Al_3_Sc. Considering the cost of Sc, the doped Al_3_Sc was more valuable for industrial application.

**Figure 4:**
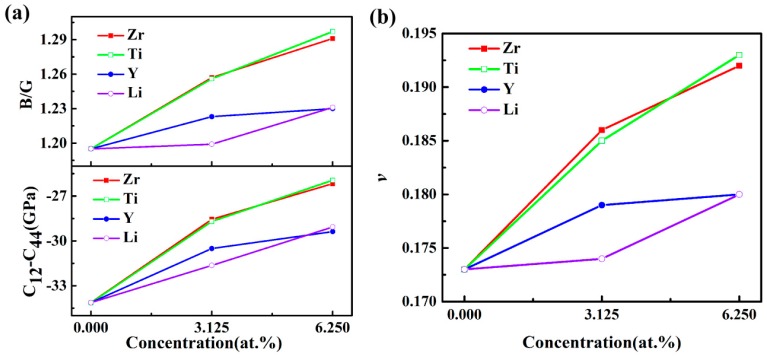
(**a**) *B*/*G* and (C_12_ – C_44_) and (**b**) ν of L1_2_-Al_3_Sc doped with element M (M = Zr, Ti, Y, and Li) as a function of the doping concentration.

The *B*/*G* ratio was used to distinguish the ductility and brittleness of compounds [38]. The greater value of *B*/*G* corresponded to a better ductility in the material. The results shown in Figure 4a indicate that the L1_2_-Al_3_Sc structure displayed a higher ductility, after doping. This conclusion could be proved by the Cauchy pressure (C_12_−C_44_) [39]. Furthermore, the *B*/*G* values for the doped Al_3_Sc increased with the elevated doping concentration, which demonstrated that a higher doping concentration enhances the ductility of Al_3_Sc. In addition, Poisson’s ratio (*v*) reflected the transverse deformation for the material [40]. It could be clearly observed that the changing trends of *ν* with doping elements and concentrations were similar to that of *B*/*G* (Figure 4). It is common knowledge that a material exhibits better ductility, when the Poisson’s ratio is large. The calculated results demonstrated that a better ductility of Al_3_Sc doped with Zr/Ti was exhibited, compared to Y/Li.

The elastic anisotropy has an important implication in engineering science, since it is highly correlated with the possibility to induce micro cracks in materials [41]. The expression of elastic anisotropy (*A*) is shown below:

(8) $$A=2C_{44}/\left(C_{11}-C_{12}\right)$$

It is noted that the material is isotropic when *A* is equal to 1. The degree to which the value of *A* deviates from 1 represents the strength of the anisotropy of the material. The *A* values of Al-Sc-M (M = Zr/Ti/Y/Li) were calculated, and the results are listed in Table 3. The results showed that Al_3_Sc had exhibited anisotropic behavior, before and after doping. Moreover, the *A* values for the doped Al_3_Sc were more deviated from 1, when the doping concentrations varied from 3.125% to 6.25%. It is worth noting that the deviation degree of Al-Sc-Li system was the largest. For example, Al_24_Sc_6_Li_2_ possessed the strongest anisotropy.

### 3.3. Debye Temperature

Debye temperature (*Θ_D_*) is a fundamental parameter for the material’s thermodynamic properties. It is correlated with many physical properties (i.e., specific heat, elastic constant, and melting temperature). The *Θ_D_* value of a solid can usually be calculated from the sound velocity. *Θ_D_* is related to the modulus of elasticity in Anderson’s model [42]. Then, *Θ_D_* is defined as [43]:

(9) $$\Theta_{D}=\frac{h}{k}{\left[\frac{3n}{4\pi }\left(\frac{N_{A}\rho }{M}\right)\right]}^{1/3}\nu_{m}$$

(10) $$\upsilon_{m}={\left[\frac{1}{3}\left(\frac{2}{\upsilon_{t}{}^{3}}+\frac{1}{\upsilon_{l}{}^{3}}\right)\right]}^{-1/3}$$

(11) $$\upsilon_{t}=\sqrt{\frac{G}{\rho }}$$

(12) $$\upsilon_{l}=\sqrt{\frac{3B+4G}{3\rho }}$$

where *h*, *k*, *N_A_*, *n*, *ρ*, *M*, $\upsilon_{m}$, $\upsilon_{t}$, and $\upsilon_{l}$ denote Planck’s, Boltzmann’s and Avogadro’s constants, total number of atoms, density, molecular weight, average sound velocity, transverse sound velocity, and longitudinal sound velocity, respectively. 

**Figure 5:**
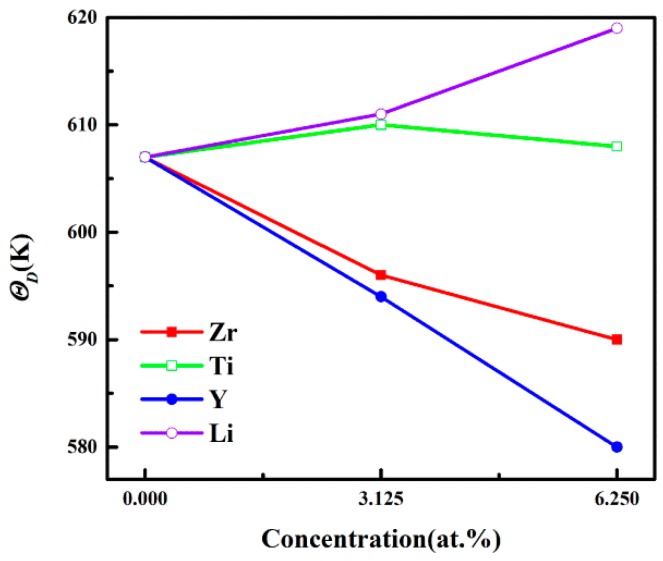
The *Θ_D_* values of L1_2_-Al_3_Sc doped with element M (M = Zr, Ti, Y, and Li) as a function of the doping concentration.

In the Debye theory, *Θ_D_* is the temperature of a crystal’s highest normal mode of vibration. That is, the highest temperature can be achieved due to a single normal vibration. It is well-known that a higher *Θ_D_* corresponds to a better thermal conductivity of a material. Figure 5 describes *Θ_D_* of Al_3_Sc, at different doping elements and concentrations. The *Θ_D_* values of the Al-Sc-Li structure increased with an increasing concentration. On the contrary, the *Θ_D_* values of Al-Sc-M (M = Zr/Y) decrease with the increasing concentration. For the Al-Sc-Ti structure, the *Θ_D_* values were a little higher than Al_3_Sc. However, the *Θ_D_* had decreased slightly, when the concentration increased from 3.125% to 6.25%. The higher *Θ_D_* values of the Al-Sc-Li structure indicated that their thermal conductivities were better, compared to other structures.

Based on the above calculation results, it was noticeable that the species and concentrations of the doped elements had intensive impacts on the mechanical properties of Al_3_Sc. In order to obtain high performance compounds, it was important to select appropriate doped element and concentration. The mechanical properties of Al_3_Sc before and after doping were intensively compared. It was demonstrated that doping Ti/Zr could better optimize the performances of the Al_3_Sc structure. This conclusion was consistent with previous reports [14,17,23]. Furthermore, the mechanical properties of Al-Sc-Ti were slightly better than that of Al-Sc-Zr. This discrepancy with the result, measured through experiments, might have arisen from a different temperature. It is worth noting that the experimental measurement was generally operated at 573 K, whereas, this work was completed at 0 K. To be more important, the structure with a higher doping concentration (6.25%) had a higher performance over the lower doping concentration (3.125%).

### 3.4. Electronic Properties

**Figure 6:**
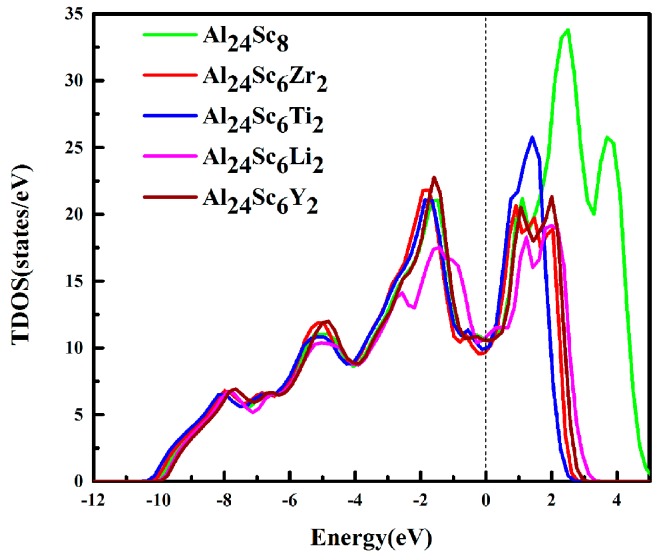
Total density of states (TDOSs) of the Al_3_Sc and Al_24_Sc_6_M_2_ (M = Sc, Zr, Ti, Li and Y) alloys.

To gain a better understanding of the doping effects at the electronic level, the total densities of states (TDOS) and partial densities of states (PDOS) were calculated in this work. The calculated TDOS are shown in Figure 6. The Fermi energy level (*E_f_*) represented by a dotted line was set to zero. It could be clearly seen that the TDOS of the structure was not zero at the Fermi level, indicating that the structures had good metallic properties [2]. Meanwhile, the TDOS of Al_24_Sc_6_M_2_ (M = Zr/Ti/Y/Li) were expensed in the energy scales.

The structures of Al_3_Sc, before and after doping, had a very wide pseudogap around *E_f_*, implying that Al-Sc-M intermetallic compounds had strong covalent bonds, indicating that it had a good stability [44]. Moreover, the widths of the pseudogap for Al_3_Sc and Al-Sc-M (M = Zr, Ti, Y, and Li) structures were 2.65 eV, 2.74 eV, 2.53 eV, 2.59 eV, and 2.23 eV, respectively. It was indeed seen that Al_24_Sc_6_Zr_2_ was slightly wider than the pseudogap of Al_3_Sc, while that of Al_24_Sc_6_M_2_ (M = Ti/Y/Li) was slightly narrower. This proved that Al_24_Sc_6_Zr_2_ had a stronger covalent bond, which was the most stable [13,14,23]. However, the stability of M (M = Ti/Y/Li) doped Al_3_Sc was weakened. This was consistent with the calculation of the formation energy in Table 1. 

**Figure 7:**
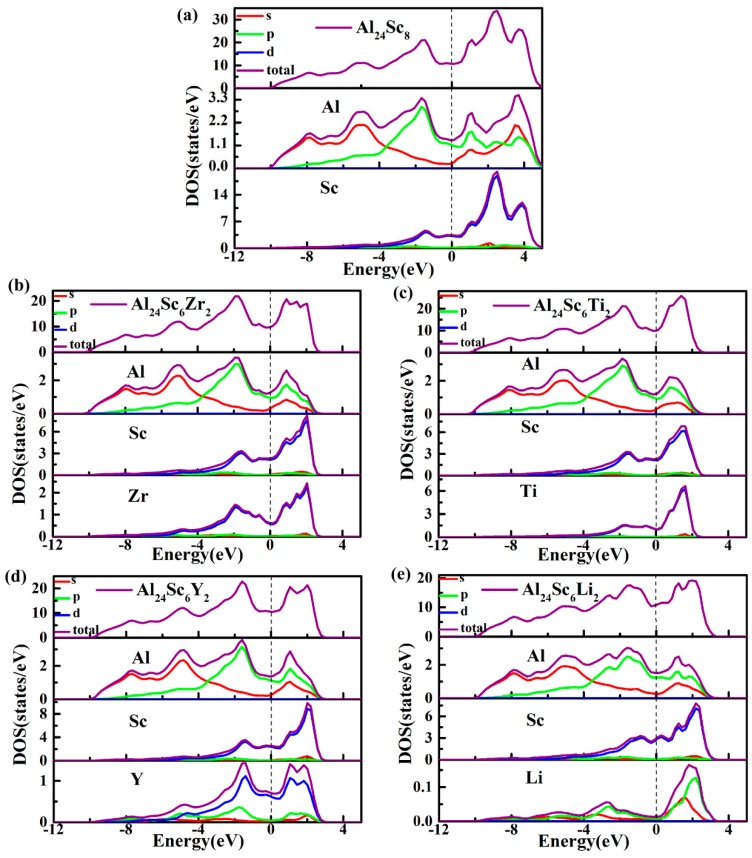
Total density of states (TDOSs) and Partial density of states (PDOSs) of (**a**) Al_3_Sc and (**b**–**e**) Al_24_Sc_6_M_2_ (M = Zr, Ti, Y, and Li) alloys. TDOSs for different structures and different elements are represented by purple lines. PDOSs for different orbitals are denoted by *s* in red, *p* in green and *d* in blue.

The reason for this consequence, the Zr-*d* orbital provided more valence electrons to hybridize with the Al-*p* orbital than the Sc-*d* orbital, while the Ti-*d* orbital provided fewer valence electrons. At the same time, there was a stronger *d*-*d* bond interaction between the Sc and Zr atoms, which effectively enhanced the ductility of the material [45]. For the Al_24_Sc_6_Li_2_ structure, the Li atom replaced the Sc atom, and reduced the *p*-*d* hybridization. The number of bonding electrons per atom in Al_3_Sc and Al-Sc-M (M = Zr, Ti, Y, and Li) structures in the low energy region are shown in Figure 7, which were 2.94, 3.022, 2.994, 2.943, and 2.811, accordingly (energy range between−12 eV and Fermi levels). It is well-known that a higher number of bonding electrons implies an increased structural stability [34,46]. Thus, a stronger electron interaction should occur in Al_24_Sc_6_Zr_2_, and Al_24_Sc_6_Zr_2_ should have a larger structural stability. It was considered that as Zr had more valence electrons, it resulted in stronger electron interactions between the Zr-*d* orbital and the Al-*p* orbital, as well as between (Sc, Zr)-*d* [45,47].

To further illustrate the contribution of each atomic orbital to TDOS, the PDOS of each atom were calculated, as shown in Figure 7. The main bonding peaks of Al_24_Sc_6_M_2_ were predominantly derived from the Al-*s* and Al-*p* orbitals, in the energy range between −12 eV and −4 eV (Figure 7b–e), making the TDOS of Al_24_Sc_6_M_2_ almost coincident with the Al_3_Sc.

It could be clearly observed that, from −4 eV to 5 eV, the TDOS was mainly contributed by the strong hybridization of the Al(Li)-*p* and Sc(Zr/Ti/Y)-*d* orbitals, and a small contribution of Al-*s* was also observed. Additionally, there was a large overlap in the entire energy range, leading to a strong *pd* hybridization. The pseudogap was generated by the hybridization of Al-*p* and M-*d* (M = Sc, Zr, Ti, Y). In other words, there was a strong covalent bond in the Al-Sc-M (M = Zr, Ti, Y, and Li) structure. In addition, the PDOS of Zr/Ti/Y-*d* orbitals were different from the Sc-*d* orbital, suggesting that the doping elements should have taken effect on the TDOS. Below the Fermi level, the peaks of Zr-*d* orbital moved toward to the lower energy level (Figure 7b), contributing to the enhancement of the bonding states of Al_24_Sc_6_Zr_2_. Regardless, the Sc-*d* orbital of Al_24_Sc_6_Li_2_ moved toward the higher level (Figure 7e). Moreover, the magnitudes of Li-*s*/*p* orbitals were tiny, compared to the other atomic orbitals. This implied that there was a weaker covalent bond in Al_24_Sc_6_Li_2_ for the subdued hybridization between Li and Sc atoms.

**Figure 8:**
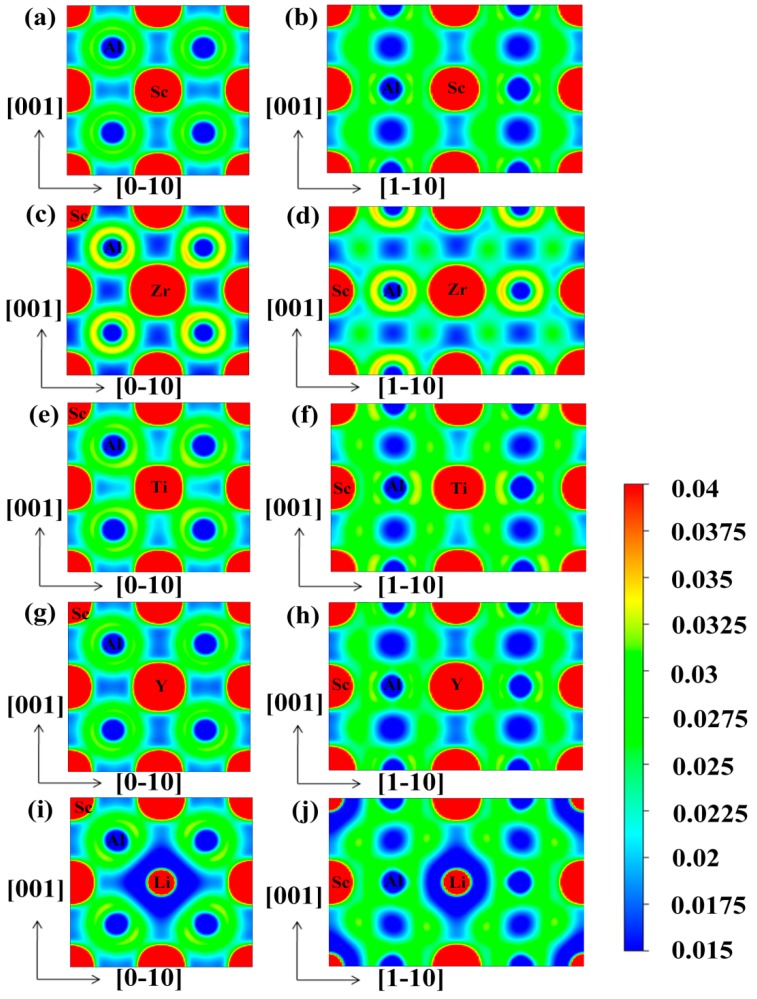
The electron density contour plots (Unit: e/Å^3^) on the (100) and (110) planes for L1_2_-Al_3_Sc (**a**,**b**), L1_2_-Al_24_Sc_6_Zr_2_ (**c**,**d**), L1_2_-Al_24_Sc_6_Ti_2_ (**e**,**f**), L1_2_-Al_24_Sc_6_Y_2_ (**g**,**h**), and L1_2_-Al_24_Sc_6_Li_2_ (**i**,**j**).

For a deeper insight into the atomic bonding of the doped structures, the valence electron density distribution were also investigated. For example, the charge densities on the (100) and (110) planes, for each cell, are shown in Figure 8, in which the contour lines are plotted from 0.015 to 0.04 e/Å^3^ with 0.0025 e/Å^3^ interval.

Figure 8a,b display the charge distribution of pure Al_3_Sc on the (100) and (110) planes, respectively. It was clearly observed that the charge densities of the neighboring Al-Al, Al-Sc, and Sc-Sc had overlaps, especially between the Al-Sc, indicating that there were strong covalent bonds in Al_3_Sc. Moreover, these covalent bonds were mainly generated by the hybridization between Al-*p* and Sc-*d* orbitals. Compared with (110) plane, the charge distribution between the neighboring Al-Sc on the (100) surface was weak, which proved that the covalent bond on the (110) plane was stronger, leading to a brittle fracture. This was caused by the difference in the local symmetries between the (100) and (110) plane. This feature was consistent with previous reports [36]. The charge densities of Al_24_Sc_6_Zr_2_ (100) and the (110) planes are shown in Figure 8c,d, respectively. The overlap of the charge density between the neighboring Al-Zr was increased, which meant that the covalent bond was enhanced. Moreover, Zr was slightly less electronegative than Sc. On this account, Al-Zr exhibited weaker ionic bond properties. This property could be analyzed by the Bader charge [48,49]. The calculation results showed that 0.38 electrons were transferred from Sc to Al in Al_3_Sc. Nevertheless, the charge transferred from (Sc, Zr) to Al was reduced to 0.34, and the charge distribution on the Sc atom remained unchanged. However, the bonding difference between (100) and (110) planes was decreased, which was profitable for the improvement of the ductility of Al_24_Sc_6_Zr_2_ [24]. The charge distributions of the Al_3_Sc doped with Ti and Y are shown in Figure 8e–h, accordingly. In contrast to the Al_24_Sc_6_Zr_2_, the overlaps of charge density between Al-Ti/Y, Sc-Ti, and Sc-Sc were reduced, implying that the covalent bonds were weakened. The charge density distributions of Al_3_Sc doped with Li are shown in Figure 8i,j. It was observed that the charge density overlap between Sc-Li decreased, indicating that Sc-Li exhibited a weaker covalent bond, which was mainly contributed by the hybridization of the Sc-*d* and Li-*p* states. 

## 4. Conclusions

In order to explore the effects of the doped elements (M) on the mechanical properties of Al_3_Sc, both, the structural stability and mechanical properties of Al_3_Sc with different doping elements and concentrations, in combination with the influence of the higher doping concentration on the electronic properties of Al_3_Sc were systematically investigated using the first-principles methods. Based on the results of this study, the following conclusions could be deduced. First, it was observed that the Al_3_Sc structure could be stable after doping. For instance, Al-Sc-Zr had the highest stability, and Al_24_Sc_6_Zr_2_ performed better on stability, compared to Al_24_Sc_7_Zr. On the other hand, Al-Sc-M (M = Ti, Y, Li) reduced the stability of Al_3_Sc, and Al_24_Sc_6_M_2_ performed worse on stability, compared with Al_24_Sc_7_M. The calculated elastic constants of Al_3_Sc, before and after doping, showed its mechanical stability. Moreover, the calculated *B*/*G* results revealed that the doped Al_3_Sc with a higher concentration exhibited a better ductility, especially, when doped with Zr and Ti. It was noted that, the calculated results of the elastic modulus *B*, *G*, *E*, and *ν* suggested that both Al_24_Sc_6_Zr_2_ and Al_24_Sc_6_Ti_2_ displayed better mechanical properties. Additionally, the TDOS and PDOS analyses indicated that the doped Al_3_Sc had a pseudogap and a strong covalent bonding, which was due to the strong *pd* state hybridization. Among them, the maximum pseudogap existed in Al_24_Sc_6_Zr_2_, indicating its best stability. This conclusion was consistent with the calculated formation enthalpy. Ultimately, the obtained results could provide an important theoretical basis for a wide application of the Al-Sc alloy.

## Figures and Tables

**Table 1:** The crystal parameters (Å), density (*ρ*/g·cm^−3^), and heat of formation (Δ*H_f_*/eV·atom^−1^) of L1_2_-Al_3_Sc_1–*x*_M*_x_* at the ground state.

Structures	*a*/Å	*ρ*/g·cm^−3^	Δ*H_f_*/eV·atom^−1^
Al_3_Sc	4.107	3.108	−0.443
Al_24_Sc_7_Zr	8.210	3.160	−0.449
Al_24_Sc_6_Zr_2_	8.211	3.298	−0.456
Al_24_Sc_7_Ti	8.180	3.064	−0.435
Al_24_Sc_6_Ti_2_	8.144	3.155	−0.427
Al_24_Sc_7_Y	8.252	3.106	−0.438
Al_24_Sc_6_Y_2_	8.291	3.19	−0.432
Al_24_Sc_7_Li	8.201	2.917	−0.396
Al_24_Sc_6_Li_2_	8.193	2.812	−0.35

**Table 2:** Calculated elastic constants Cij for L1_2_-Al_3_Sc_1–*x*_M*_x_* at the ground state.

Structures	C_11_ (GPa)	C_12_ (GPa)	C_44_ (GPa)	C_11_ − C_12_ (GPa)	C_12_ − C_44_ (GPa)
Al_3_Sc	183.99	37.81	71.94	146.18	−34.13
Exp. [31]	183	46	68	137	−22
DFT [15]	180.67	40.62	72	140.05	−31.38
Al_24_Sc_7_Zr	181.2	40.89	69.44	140.31	−28.55
Al_24_Sc_6_Zr_2_	183.93	42.9	69.07	141.03	−26.17
Al_24_Sc_7_Ti	181.66	40.88	69.58	140.79	−28.7
Al_24_Sc_6_Ti_2_	186.41	43.325	69.26	143.09	−25.94
Al_24_Sc_7_Y	175.77	38.08	68.59	137.69	−30.51
Al_24_Sc_6_Y_2_	171.96	38.55	67.93	133.41	−29.38
Al_24_Sc_7_Li	167.18	38.05	69.69	129.37	−31.64
Al_24_Sc_6_Li_2_	163.42	39.34	68.41	124.08	−29.07

Note: Exp. represents the reported “experimental results”.

**Table 3:** Elastic moduli *B*, *G*, *E*, ***ν***, *B*/*G*, *H*, *A* and *Θ_D_* for L1_2_-Al_3_Sc_1–*x*_M*_x_*, at the ground state.

Structures	*B* (GPa)	*G* (GPa)	*E* (GPa)	*ν*	*B*/*G*	*A*	*H* (GPa)	*Θ_D_* (K)
Al_3_Sc	86.53	72.4	169.84	0.173	1.195	0.98	15.78	607
Exp. [31]	91.7	71.7	170.63	0.201	1.28	0.99		
DFT [15]	87.3	71.2	167.94	0.179	1.23	1.03		
Al_24_Sc_7_Zr	87.66	69.73	165.34	0.186	1.257	0.99	14.59	596
Al_24_Sc_6_Zr_2_	89.91	69.64	166.06	0.192	1.291	0.98	14.3	590
Al_24_Sc7Ti	87.81	69.91	165.74	0.185	1.256	0.98	14.68	610
Al_24_Sc_6_Ti_2_	91.02	70.16	167.48	0.193	1.297	0.97	14.37	608
Al_24_Sc_7_Y	83.98	68.64	161.93	0.179	1.223	0.99	14.7	594
Al_24_Sc_6_Y_2_	83.02	67.44	159.21	0.18	1.23	1.02	14.39	580
Al_24_Sc_7_Li	81.09	67.64	158.77	0.174	1.199	1.07	14.61	611
Al_24_Sc_6_Li_2_	80.97	65.78	155.43	0.18	1.231	1.10	14.05	619

Notes: *A* is defined for elastic anisotropy, and *Θ_D_* is for Debye temperature.
